# Handgrip strength is inversely associated with augmentation index in patients with type 2 diabetes

**DOI:** 10.1038/s41598-023-28392-8

**Published:** 2023-01-20

**Authors:** Hidetaka Hamasaki, Hidekatsu Yanai

**Affiliations:** 1 Hamasaki Clinic, 2-21-4 Nishida, Kagoshima, 890-0046 Japan; 2 Department of Diabetes, Endocrinology and Metabolism, National Center for Global Health and Medicine Kohnodai Hospital, Chiba, Japan

**Keywords:** Endocrinology, Medical research

## Abstract

Handgrip strength (HGS) is a measure of overall skeletal muscle strength and is used to identify risks for cardiovascular disease and mortality. Furthermore, HGS is an indicator of arterial stiffness that leads to atherosclerotic cardiovascular disease. This study aimed to examine the relationship between HGS and augmentation index (AIx) in patients with type 2 diabetes. A cross-sectional study was conducted to examine patients with type 2 diabetes whose HGS and AIx were measured in our hospital. AIx was measured noninvasively using an applanation tonometer, and multiple regression analyses were conducted to assess the independent relationship between HGS and AIx. This study included 404 patients. After adjusting for age, gender, body mass index, duration of diabetes, smoking and exercise habit, biochemical parameters, and physiological parameters related to arterial stiffness, HGS was found to be independently and inversely associated with AIx (β = − 0.270, *p* = 0.006). HGS was independently and inversely associated with AIx in patients with type 2 diabetes. Patients with diminished HGS should be subjected to intensive exercise therapy for reducing the risk of arterial stiffness and cardiovascular disease.

*Trial registration*: UMIN000023010.

## Introduction

Type 2 diabetes (T2DM) has become a pandemic, which is a serious problem worldwide. Patients with T2DM show lower cardiorespiratory fitness^1,2^ and muscle strength^3^ than those without. A 10-year follow-up of the Look AHEAD (Action for Health in Diabetes) study has shown that low cardiorespiratory fitness is associated with an increased risk of atherosclerotic cardiovascular disease (hazard ratio (HR) = 1.94; 95% confidence intervals (CI), 1.12–3.35) in patients with T2DM^4^. On the other hand, cardiorespiratory fitness is positively associated with muscle strength measured by handgrip test in women^5^. Albin et al.^6^ reported that individuals with high cardiorespiratory fitness and strong handgrip strength (HGS) had a lower prevalence ratio of arterial stiffness (0.46; 95% CI, 0.25–0.85) than those with low cardiorespiratory fitness and weak HGS. Therefore, HGS may be an indicator for arterial stiffness that leads to atherosclerotic cardiovascular disease. HGS is a simple method for evaluating overall skeletal muscle strength and is also a useful tool for identifying risks for T2DM^7^, cardiovascular disease (CV), hospitalization, and mortality^8,9^, which provides important information on a variety of health outcomes in clinical practice.

The augmentation index (AIx) is a measure of systemic arterial stiffness and risk factor for CV^10^. AIx is considered a vascular parameter showing the deleterious effect on cardiac workload of increased wave reflection related to arterial stiffness^11^. Previous studies have reported that AIx is higher in subjects with diabetes^12,13^ and hypercholesterolemia^14^, and predicts myocardial ischemic threshold in patients with coronary artery disease^15^. Moreover, a 10% increase in AIx is associated with all-cause mortality (HR = 1.51; 95% CI, 1.23–1.86) and CV mortality (HR = 1.48; 95% CI, 1.16–1.90) in patients with end-stage renal failure^16^. Not brachial-ankle pulse wave velocity (PWV) but heart rate-adjusted AIx is reduced by hemodyalysis, suggesting that AIx reflects large artery stiffness more precisely than PWV^17^.

To date, there have been several studies that examine the association between HGS and AIx; however, the results are controversial^18,19^ and few studies have investigated it in a T2DM population. If HGS has a significant relationship with AIx in patients with T2DM, HGS could also be a useful indicator for large artery stiffness. Thus, the present study aims to examine the associations of HGS with AIx in patients with T2DM.

## Materials and methods

### Study design and subjects

We conducted a cross-sectional study in patients with T2DM who were treated at the National Center for Global Health and Medicine Kohnodai Hospital. A total of 406 individuals whose HGS and AIx were measured at the medical examination (the same consultation day) were included. Trained technicians at the Clinical Research Center of the National Center for Global Health and Medicine at Kohnodai Hospital asked participants at the outpatient clinic about the subjects’ history of CV events, smoking habit, and exercise habit such as resistance training and walking. Patients with type 1 diabetes (n = 2) were excluded. The opt-out method of obtaining informed consent was used as this study was a retrospective analysis of the existing data. The study protocol was approved by the Medical Ethics Committee of the National Center for Global Health and Medicine (Reference No. NCGM-G-002052), and the study was performed in accordance with the Declaration of Helsinki.

### Anthropometric and physiological measurements

Height was measured using a rigid stadiometer (TTM stadiometer; Tsutsumi Co., Ltd., Tokyo, Japan). Weight was measured using calibrated scales (AD-6107NW; A&D Medical Co., Ltd., Tokyo, Japan). Body mass index (BMI) was calculated as body weight in kilograms divided by the square of body height in meters. Waist circumference was measured at the umbilical level at the end of exhalation in a standing position. HGS was measured using a Smedley analog hand dynamometer (No. 04125; MIS, Tokyo, Japan) twice, using both hands in a standing position. We utilized the average HGS in kilograms. Peripheral arterial stiffness was examined by measuring the PWV using a pulse pressure analyzer (BP-203RPE; Nihon Colin, Tokyo, Japan), and central systolic blood pressure (cSBP) and the AIx corrected for a heart rate of 75 bpm (AIx_75_) were measured noninvasively using an applanation tonometer (HEM-9000AI; Omron Co., Ltd, Tokyo, Japan). Patients put their left forearm on the elbow placement board of the device in a sitting position. Left radial pulse waves and left brachial blood pressures were automatically recorded. After recording the radial and aortic pulse waveforms, cSBP was automatically estimated using a linear equation by the device^20^.

### Blood examination

We measured plasma glucose (PG), hemoglobin A1c (HbA1c), B-type natriuretic peptide (BNP), serum high-density lipoprotein cholesterol (HDL-C), and low-density lipoprotein cholesterol (LDL-C) levels. Recent studies identified LDL-C-to-HDL-C ratio (LDL-C/HDL-C) as a better biomarker to predict both atherosclerosis progression and CV than HDL-C or LDL-C alone^21–23^. Thus, we calculated LDL-C/HDL-C as an index for arterial sclerosis. We also calculated estimated glomerular filtration rate (eGFR) using the revised equation adjusted for the Japanese population^24^.

### Sample size

Sample size calculation was performed using G*Power (https://www.psychologie.hhu.de/arbeitsgruppen/allgemeine-psychologie-und-arbeitspsychologie/gpower). Effect size was set at 0.02 according to Cohen’s definition^25^. The number of predictors was assumed to be 10 (age, gender, BMI, duration of diabetes, exercise habit, HbA1c, LDL-C/HDL-C, eGFR, BNP, and cSBP), the one-tailed alpha level is 0.025, and the sample size calculation indicated that at least 395 subjects were needed for a power of 0.8. This suggests that our sample size had sufficient power to detect the association between HGS and AIx.

### Statistical analysis

Statistical analyses were performed using SPSS version 25 (IBM Co., Ltd., Chicago, IL). All numerical variables are expressed as the mean ± standard deviation. Pearson’s correlation coefficient was calculated to analyze the associations of HGS with the clinical data. Mann–Whitney U test or Student’s t test, depending on the normality test results (Shapiro–Wilk test), was performed to detect significant differences between men and women, as appropriate. χ2 test was also performed to identify significant differences in categorical variables between men and women. Multiple regression analysis was performed to test independent associations between HGS (independent variable) and AIx_75_ (dependent variable). The regression modeling was performed in three steps with age, gender, BMI, smoking habit, and duration of diabetes being included as possible confounders in the model 1, and further adjustments for exercise habit, HbA1c, and LDL-C/HDL-C (model 2), and finally, further adjustments for eGFR, BNP and cSBP (model 3). In addition, *p* values of < 0.05 determined by performing a two-sided test were considered as indicating statistical significance.

## Results

This study enrolled 404 patients (231 men and 173 women) with T2DM. The mean age and duration of diabetes of study participants were 63.1 ± 14.3 and 11.2 ± 11.8 years, respectively. The mean BMI was 25.7 ± 5.7 kg/m^2^. Sixty patients (14.9%) had a history of CV. Eighty-six patients (21.3%) were current smokers. Two-hundred and three patients (50.2%) engaged in habitual exercise. Two-hundred and sixty-seven patients (66.1%) received glucose-lowering drugs, one hundred and twenty-three patients (30.4%) received statins, one hundred and twenty-four patients (30.7%) received renin-angiotensin system inhibitors, and fifty-eight patients (14.4%) received antiplatelet drugs. Demographic, anthropometric, and clinical data are listed in Table 1.

**Table 1:** Clinical characteristics of the subjects.

Demographics and anthropometric data	
n	404
Age (years)	63.1 (14.3)
Gender (male/female)	231/173
Smoking habit (yes/no)	86/318
Number of cigarettes (cigarettes/day)	10.7 (15.4)
Exercise habit (yes/no)	203/201
Duration of diabetes (years)	11.2 (11.8)
History of CV (yes/no)	60/344
Height (cm)	160.9 (9.5)
Weight (kg)	67.1 (17.6)
BMI (kg/m^2^)	25.7 (5.7)
Waist circumference (cm)	92.7 (14.1)
Handgrip strength (kg)	23.9 (9.7)

Physiological and biochemical data	
Plasma glucose (mg/dL)	177.9 (78.5)
HbA1c (%)	8.3 (2.1)
Serum HDL-C (mg/dL)	50.3 (13.8)
Serum LDL-C (mg/dL)	113.6 (38.0)
LDL-C/HDL-C	2.43 (1.01)
Plasma BNP (pg/mL)	41.0 (121.9)
eGFR (mL/min/1.73m^2^)	75.4 (27.4)
PWV (cm/s)	1712.8 (436.0)
cSBP (mmHg)	132.0 (22.1)
AIx_75_	76.2 (13.0)

Medications	Number of subjects
Glucose-lowering drugs	267
Insulin	52
Statins	123
Lipid-lowering drugs except statins	42
RASi	124
Antihypertensive drugs except RASi	138
Antiplatelet drugs	58
Anticoagulant drugs	12

Data are represented as the mean (SD) except for the number of subjects, gender, smoking habit, exercise habit, and history of CV. CV, cardiovascular disease; BMI, body mass index; HbA1c, hemoglobin A1c; HDL-C, high-density lipoprotein cholesterol; LDL-C, low-density lipoprotein cholesterol; BNP, B-type natriuretic peptide; eGFR, estimated glomerular filtration rate; PWV, brachial-ankle pulse wave velocity; cSBP, central systolic blood pressure; AIx_75_, augmentation index corrected for a heart rate of 75 bpm; RASi, Renin-angiotensin system inhibitors.

Table 2 shows the difference between men and women in clinical data. Women had higher HDL-C and AIx_75_; on the other hand, women had lower HGS than men. The number of smokers and cigarettes per day were larger in men than in women.

**Table 2:** Comparison of clinical data of men and women.

	Men	Women	*p*
Age (years)	62.7 (14.7)	63.7 (13.8)	0.46
Smoking habit (yes/no)	67/165	19/153	< 0.001
Number of cigarettes (cigarettes/day)	15.1 (17.0)	4.8 (10.5)	< 0.001
Exercise habit (yes/no)	120/112	83/89	0.48
Duration of diabetes (years)	12.2 (12.2)	10.0 (11.1)	0.06
History of CV (yes/no)	38/193	22/151	0.33
Height (cm)	167.0 (6.5)	152.8 (6.4)	< 0.001
Weight (kg)	71.3 (17.8)	61.4 (15.8)	< 0.001
BMI (kg/m^2^)	25.4 (5.4)	26.2 (6.0)	0.21
Waist circumference (cm)	92.4 (13.5)	93.2 (14.9)	0.58
Handgrip strength (kg)	28.8 (8.5)	17.4 (7.1)	< 0.001
Plasma glucose (mg/dL)	180.2 (80.7)	175.0 (75.6)	0.52
HbA1c (%)	8.4 (2.1)	8.3 (2.1)	053
Serum HDL-C (mg/dL)	48.3 (12.6)	53.0 (15.0)	0.001
Serum LDL-C (mg/dL)	113.2 (38.3)	114.0 (37.9)	0.86
LDL-C/HDL-C	2.51 (1.01)	2.32 (0.99)	0.13
Plasma BNP (pg/mL)	45.7 (153.2)	34.8 (58.6)	0.50
eGFR (mL/min/1.73m^2^)	75.3 (27.3)	75.6 (27.7)	0.91
PWV (cm/s)	1742.7 (441.4)	1673.9 (427.0)	0.12
cSBP	131.4 (22.7)	132.8 (21.3)	0.53
AIx_75_	72.6 (13.3)	81.0 (10.9)	< 0.001

Data are represented as the mean (SD) except for smoking habit, exercise habit, and history of CV. CV, cardiovascular disease; BMI, body mass index; HbA1c, hemoglobin A1c; HDL-C, high-density lipoprotein cholesterol; LDL-C, low-density lipoprotein cholesterol; BNP, B-type natriuretic peptide; eGFR, estimated glomerular filtration rate; PWV, brachial-ankle pulse wave velocity; cSBP, central systolic blood pressure; AIx_75_, augmentation index corrected for a heart rate of 75 bpm.

**Figure 1:**
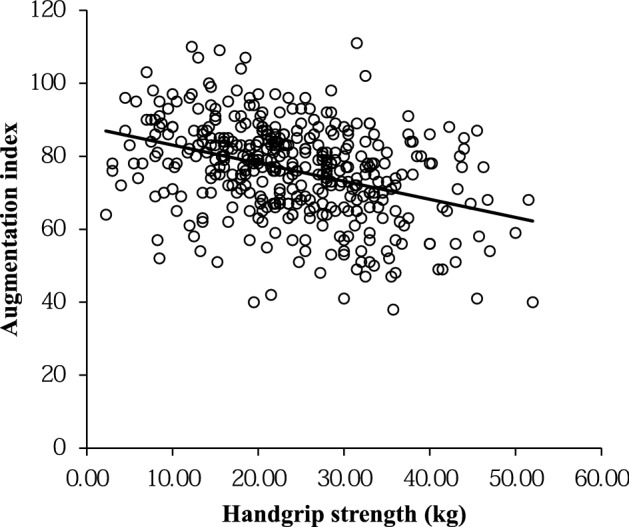
Correlation between handgrip strength and augmentation index.

HGS was inversely correlated with age (r = − 0.390, *p* < 0.001), duration of diabetes (r = − 0.105, *p* = 0.037), HDL-C (r = − 0.172, *p* = 0.001), PWV (r = − 0.184, *p* < 0.001), AIx_75_ (r = − 0.372, *p* < 0.001) (Fig. 1), and plasma BNP levels (r = − 0.129, *p* = 0.048). In contrast, HGS was positively correlated with BMI (r = 0.221, *p* < 0.001), waist circumference (r = 0.188, *p* < 0.001), eGFR (r = 0.110, *p* = 0.028), and LDL-C/HDL-C (r = 0.153, *p* = 0.013).

The adjusted associations between HGS and AIx_75_ are presented in Table 3 (model 1–3). After adjustment for age, gender, BMI, duration of diabetes, smoking habit, exercise habit, HbA1c, LDL-C/HDL-C, eGFR, cSBP, and BNP, HGS was still inversely associated with AIx_75_ (β = − 0.270, *p* = 0.006).

**Table 3:** Associations between handgrip strength and augmentation index in the multiple regression models.

	Model 1		Model 2		Model 3	
	β	*p*	β	*p*	β	*p*
Age	0.336	< 0.001	0.322	< 0.001	0.206	0.042
Gender						
Men	− 0.243	< 0.001	− 0.205	0.004	− 0.176	0.045
Women (reference)	–	–	–	–	–	–
Duration of diabetes	− 0.147	0.002	− 0.227	< 0.001	− 0.215	0.005
BMI	− 0.027	0.59	− 0.016	0.80	− 0.060	0.44
Smoking habit	0.088	0.064	0.158	0.008		
Exercise habit	–	–	0.067	0.24	0.078	0.25
HbA1c	–	–	− 0.046	0.42	− 0.028	0.70
LDL-C/HDL-C	–	–	0.050	0.40	− 0.056	0.43
eGFR	–	–	–	–	0.125	0.15
BNP	–	–	–	–	0.060	0.41
cSBP	–	–	–	–	0.342	< 0.001
HGS	− 0.147	0.019	− 0.205	0.008	− 0.270	0.006

BMI, body mass index; HbA1c, hemoglobin A1c; HDL-C, high-density lipoprotein cholesterol; LDL-C, low-density lipoprotein cholesterol; BNP, B-type natriuretic peptide; eGFR, estimated glomerular filtration rate; cSBP, central systolic blood pressure; HGS, handgrip strength. Model 1: adjusted for age, gender, BMI, duration of diabetes, and smoking habit. Model 2: adjusted for age, gender, BMI, duration of diabetes, smoking habit, exercise habit, HbA1c, and LDL-C/HDL-C. Model 3: age, gender, BMI, duration of diabetes, smoking habit, exercise habit, HbA1c, LDL-C/HDL-C, eGFR, BNP, and cSBP.

Furthermore, HGS was significantly lower in subjects with a history of CV than in subjects without (20.9 ± 10.4 kg vs. 24.4 ± 9.5 kg, *p* = 0.009).

## Discussion

We demonstrated that HGS was inversely associated with AIx_75_ in patients with T2DM. The association was observed independently of age, gender, BMI, duration of diabetes, smoking habit, exercise habit, and biochemical and physiological parameters related to arterial stiffness. To our knowledge, this is the first study to report a significant association between HGS and AIx in a T2DM population. Lima-Junior et al.^18^ already reported significant associations between HGS and arterial stiffness including AIx in hypertensive patients; however, the findings of this study will provide important insight into considering why the measurement of HGS can identify the risk of CV events and mortality in patients with T2DM.

AIx measures the augmentation of central aortic pressure by a pulse wave reflection^26^ and indicates the degree of arterial stiffness of both elastic and muscular arteries. Recently, König et al.^27^ have reported that a decrease in HGS of 5 kg is associated with an increase in PWV of 0.08 m/s after adjustment for cofounders. This study suggests that arterial stiffness link decrease in muscle strength and/or mass and increased risk of cardiovascular mortality. Arterial stiffness may play a key role in the occurrence of CV events in patients with T2DM whose HGS is low as we previously reported^9^. Acute handgrip exercises increase AIx and cSBP which is more strongly related to CV than brachial blood pressure in healthy individuals^28^, while AIx decreases after maximal exercise in T2DM patients who usually have impaired cardiac function^29^. The effect of acute exercise on arterial stiffness is different between healthy individuals and patients with T2DM^30^; therefore, we should also be careful to interpret the association between HGS and AIx in patients with T2DM. We cannot reveal the mechanism underlying the influence of low HGS on arterial stiffness based on just the findings of this study; however, oxidative stress, endothelial dysfunction, and inflammation may contribute to both lowering muscle strength^31–34^ and the progression of arterial stiffness^35–37^ in patients with T2DM. Furthermore, current evidence suggests that there is a crosstalk between myokines and multi-organs to maintain glucose homeostasis^38^, which might mediate between skeletal muscle and the progression of arterial stiffness in patients with T2DM. Indeed, secretion of some myokines (e.g., interleukin- (IL-) 6, IL-8, IL-15, and myostatin) are altered in human skeletal muscle cells cultured from patients with T2DM^39^. A recent study showed that aerobic exercise training increased a myokine, irisin secretion, which was associated with improvement of arterial stiffness in rats with obesity^40^. To elucidate the common mechanism for decline in muscle fitness and progression of arterial stiffness, further investigations are required. However, given that muscle strength is a modifiable risk factor for CV, exercise therapy aiming to increase muscle strength can be effective for reducing the risk of atherosclerotic CV in patients with T2DM^41^.

The inverse correlation between HGS and BNP levels is intriguing. Previous studies showed that AIx was positively associated with the elevation of BNP levels in patients with paroxysmal atrial fibrillation^42^ and N-terminal pro BNP levels in patients with peripheral artery disease^43^. We also observed the positive correlation between AIx_75_ and BNP (r = 0.164, *p* = 0.012) in this study. A possible mechanism explaining this relationship is that arterial stiffness has an impact on diastolic dysfunction^44^. The progression of arterial stiffness increases pulse wave velocity generated by left ventricular contraction, which enhances the early return of wave reflection to the heart and the augmentation of central aortic pressure. As a result, the cardiac afterload increases and then may cause diastolic dysfunction and/or left ventricular hypertrophy^42^. Considering that the prevalence of left ventricular diastolic dysfunction in patient with T2DM is significantly high (35%)^45^, the relationship among HGS, arterial stiffness, and BNP that is a prognostic marker for heart failure should be further investigated. Both HGS and BNP are predictive for CV events in patients with T2DM^46^. As mentioned above, skeletal muscle has been recognized as an endocrine organ that secretes myokines with a favorable effect on metabolism^38,47^. Irisin increases myocardial cell metabolism^48^. Fibroblast growth factor 21 increases adiponectin levels, which has a cardioprotective effect, and attenuates cardiac remodeling after myocardial infarction^49^. Myokines are promising for cardioprotective effects and may be the key to reveal the effect of skeletal muscle on arterial stiffness.

The gender difference in AIx was consistent with the finding of a previous study. Rosenbaum et al.^50^ reported that AIx was 77 ± 12% in men and 86 ± 12% in women, which is approximately equal to the result of the present study. The authors argued that the change in sex hormones in postmenopausal status might affect arterial stiffening in women. Indeed, the mean age of female subjects in this study was 63.7 years, and 81.6% of the women were 50 years and older. The higher AIx_75_ in women than men can be explained by the effect of postmenopausal status on arterial stiffness^51^.

Generally, arterial stiffness measured by AIx progresses with aging^52^. In the present study, AIx_75_ was positively correlated to age (r = 0.333, *p* < 0.001); however, AIx_75_ was inversely associated with the duration of diabetes in the multiple regression models. As such, we divided study subjects by the median value of the duration of diabetes and reanalyzed the differences in clinical data between groups. Then, we found that the subjects with a long (≥ 8 years) duration of diabetes had more exercise habits (56.4% vs. 45.3%, *p* = 0.027) and lower BMI (25.1 ± 5.3 kg/m^2^ vs. 26.5 ± 6.0 kg/m^2^, *p* = 0.014) compared with subjects with a short duration of diabetes (< 8 years). No significant difference between groups in glycemic control, lipid profile, blood pressure, and the smoking habit was identified. The indicators of obesity, including BMI, have a positive relationship with arterial stiffness^53^. Excess adipose tissue is a key determinant of the development of hypertension, endothelial dysfunction, and arterial stiffness^54^. Moreover, regular exercise improves arterial stiffness measured by carotid intima-media thickness and PWV in patients with T2DM^55^. Even short-term, aerobic exercise effectively decreases arterial stiffness in radial, femoral, and carotid arteries in patients with T2DM^56,57^. Therefore, regular exercise habits and less body fat in subjects with longer duration of diabetes could contribute to a decrease AIx in this study population.

The relationship between skeletal muscle mass/strength and HDL-C levels is controversial. Pietrobelli et al.^58^ and Duran et al.^59^ reported that skeletal muscle mass was inversely correlated with HDL-C levels, on the other hand, higher HGS was related to higher HDL-C levels in a middle-aged population^60^. In this study, we observed inverse correlations of HGS with HDL-C levels and LDL-C/HDL-C. Excess adiposity is responsible for low HDL-C levels^61^; thus, obesity could probably explain this inconsistency. HGS is a useful tool for evaluating overall muscle mass; however, there is yet no consensus on how HGS should be used in clinical practice^62^. For example, there have been studies showing that not absolute but relative HGS such as HGS/BMI that takes the influence of obesity into consideration might be a better indicator for muscle weakness and the risk of cardiometabolic diseases^63,64^. Then, we re-analyzed the associations between HGS/BMI and clinical data. The associations of HGS/BMI with HDL-C (r = − 0.038, *p* = 0.45) and LDL-C/HDL/C (r = 0.011, *p* = 0.86) were not significant. In addition, the adjusted associations between HGS/BMI and AIx_75_ were as follows: model 1, β = − 0.140, *p* = 0.025; model 2, β = − 0.201, *p* = 0.009; model 3, β = − 0.223, *p* = 0.022, respectively. There were still significant and inverse associations between HGS/BMI and AIx_75_ in model 1, 2, and 3. Meanwhile, the ability of HGS to predict health outcomes may not be different between absolute HGS and relative HGS^65^. How HGS should be used in clinical studies has been disputed^8^, and further validation studies will be needed.

There are several limitations need to be addressed. First, this study is a single-center study, and the results may not be generalized to other populations. Second, the cross-sectional design cannot be used to infer causality. A prospective study is warranted to clarify the mechanisms regarding the relationship between HGS and the progression of arterial stiffness. Third, medications used to treat T2DM, hypertension, and dyslipidemia may intricately influence AIx and cardiovascular parameters. The possibility that medications affect the relationship between HGS and AIx cannot be excluded. Fourth, AIx is a surrogate marker for systemic arterial stiffness; thus, the inverse relationship between HGS and AIx_75_ may not prove the presence of large artery stiffness. However, Betge et al.^66^ reported that measuring AIx was suitable for detecting atherosclerosis as compared to undergoing coronary angiography. The lower HGS in subjects with a history of CV in the present study and the strong evidence that HGS was associated with an increased risk of CV events^8^ suggests that HGS could be a non-invasive indicator for arterial stiffness. Additionally, König et al.^27^ recently showed that low HGS and increased arterial stiffness are closely tied to each other. HGS could still be a precise measurement for arterial stiffness.

In conclusion, HGS was independently and inversely associated with arterial stiffness represented as AIx in patients with T2DM. This finding will shed a light on the mechanism behind the increased CV mortality in T2DM patients who have lower skeletal muscle mass/strength compared with healthy individuals. Although, there is yet no standard method for using HGS in clinical practice, T2DM patients who have lower HGS could be targeted by an intensive exercise therapy to reduce the risk of arterial stiffness and CV.

## Data Availability

The data that support the findings of this study are available from the corresponding author upon reasonable request.
